# Advances in the Fabrication of Antimicrobial Hydrogels for Biomedical Applications

**DOI:** 10.3390/ma10030232

**Published:** 2017-02-26

**Authors:** Carmen M. González-Henríquez, Mauricio A. Sarabia-Vallejos, Juan Rodriguez-Hernandez

**Affiliations:** 1 Departamento de Química, Matemáticas y del Medio Ambiente, Facultad de Ciencias Naturales, Universidad Tecnológica Metropolitana, P.O. Box 9845, Correo 21, Santiago 7800003, Chile; carmenmabel@gmail.com; 2 Departamento de Ingeniería Estructural y Geotecnia, Escuela de Ingeniería, Pontificia Universidad Católica de Chile, P.O. Box 306, Correo 22, Santiago 7820436, Chile; masarabi@uc.cl; 3 Departamento de Química y Propiedades de Polímeros, Instituto de Ciencia y Tecnología de Polímeros-Consejo Superior de Investigaciones Científicas (ICTP-CSIC), Juan de la Cierva 3, Madrid 28006, Spain

**Keywords:** hydrogels, antimicrobial activity, antimicrobial agents, chitosan, silver nanoparticles

## Abstract

This review describes, in an organized manner, the recent developments in the elaboration of hydrogels that possess antimicrobial activity. The fabrication of antibacterial hydrogels for biomedical applications that permits cell adhesion and proliferation still remains as an interesting challenge, in particular for tissue engineering applications. In this context, a large number of studies has been carried out in the design of hydrogels that serve as support for antimicrobial agents (nanoparticles, antibiotics, etc.). Another interesting approach is to use polymers with inherent antimicrobial activity provided by functional groups contained in their structures, such as quaternary ammonium salt or hydrogels fabricated from antimicrobial peptides (AMPs) or natural polymers, such as chitosan. A summary of the different alternatives employed for this purpose is described in this review, considering their advantages and disadvantages. Finally, more recent methodologies that lead to more sophisticated hydrogels that are able to react to external stimuli are equally depicted in this review.

## 1. Introduction

Hydrogels are three-dimensional networks based on the crosslinking of hydrophilic polymers chains. Formed either from natural or synthetic polymers, their tridimensional network is able to uptake a large amount of water within the structure, which confers to the material a soft consistency comparable to those observed in living tissues [1]. Thus, provided several characteristics, including the appropriate functionality, biocompatibility, mechanical properties and sterilizability, hydrogels are excellent candidates to be employed in biomedical applications, in particular for treatment or replacement of tissues or even organs [2]. These materials have been equally applied for other purposes including healing of chronic and traumatic wounds, surface coatings for implants, drug delivery and also for cell encapsulation and tissue engineering (TE) [3,4,5,6,7]. It is worth mentioning that, in order to mimic natural tissues, in addition to the chemical properties or the appropriated mechanical behavior, hydrogels are also required to be highly biocompatible at the cell level. For that purpose, the most recent approaches are attempting to finely tune both the biological and physical attributes of hydrogels aiming to induce specific interactions and responses from cellular systems [7]. 

**Figure 1:**
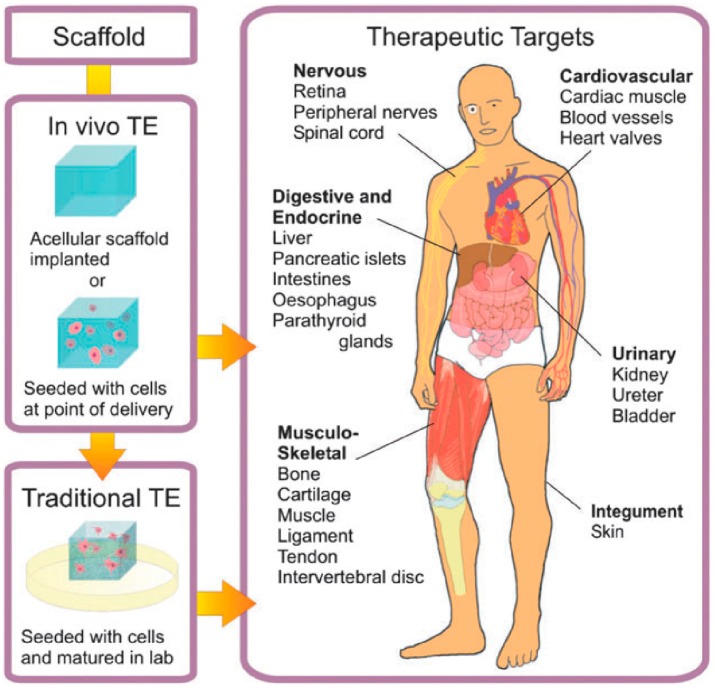
Scheme of the approaches employed for tissue engineering (TE). In traditional TE (in vitro TE), cellularized scaffolds are matured in vitro and then introduced into the patient. For in vivo TE, the scaffold is implanted directly into the patient. Reproduced with permission from [8].

Particularly extended is the use of hydrogels for TE purposes. In this case, the main objective is to generate biological tissues and organs for a variety of medical situations in which the latter present dysfunctions or are lost. As depicted by Place et al. [8] (shown in Figure 1), tissue engineering (TE) encompasses either embedding cells into a supporting structure known as a scaffold. These goals can be achieved using two different strategies. In the traditionally-employed TE, cellularized scaffolds are matured in vitro previously and then are introduced into the patient. This procedure helps with the vascularization and innervation of the inserted tissue. In contrast to this approach, in the in vivo TE approach, the scaffold is directly inserted into the patient, leaving the host cells to regenerate by themselves and can be carried out using two different types of scaffolds. On the one hand, the cells are introduced in the scaffold, before implanting it into a recipient. On the other hand, some other authors omitted this step and implanted the scaffold directly into the patient’s body. In this case, regeneration is based on the employment of native cells into the implant and the subsequent deposition of an extracellular matrix (ECM). A large variety of therapeutic targets are currently being investigated, including nervous, cardiovascular, digestive and endocrine, musculo-skeletal, urinary and integument tissues and organs. 

In order to use a particular biomaterial as a TE scaffold, several considerations should be taken into account. According to Place et al. [8], the most relevant are to fulfill the bulk mechanical and structural requirements of the target tissue and to facilitate the molecular interactions with cells that promote tissue healing. In this context, synthetic polymers, which can be obtained by using a variety of polymerization techniques, are excellent candidates. More importantly, the mechanical, chemical and physical properties of synthetic polymers are more reproducible and can be easily modulated through the variation of parameters during their formation, making them more flexible than those derived from natural materials, characteristics that are critical for the fabrication of tissue-engineering scaffolds. Polymers can be, in addition, designed to be non-toxic, inexpensive to develop and, also, can be produced using mild conditions, enabling their use in the presence of cells.

While synthetic polymers are able to satisfy most of the prerequisites for their use for TE purposes, as will be depicted throughout this review, many of the applications in which hydrogels can be employed suffer from microbial infections caused by bacteria and fungi. In effect, particularly contamination by bacteria still remains a major issue for the use of hydrogels for tissue engineering and other related biomedical applications. In order to overcome this problem, many different alternatives to fabricate antimicrobial/antifouling hydrogels that either repel or are able to kill bacteria on contact with the hydrogel surface have been developed in the last few years. Several articles, reviews and books have been recently reported describing the synthesis, properties and uses of hydrogels [9,10,11].

For example, Veiga and Schneider [3] established that, in the case of wound healing, infections can prolong the healing process, which may lead to tissue morbidity and, in the worst case, even to sepsis. Equally, infections localized at the implant-tissue interface provoke implant failure. In this situation, the implant needs to be removed and replaced for a new one, involving another surgery that can produce expensive costs, painful and highly dangerous for the patient.

In this context, hydrogels have been proposed to be an interesting alternative to fabricate antimicrobial materials, such as wound dressings, as wells as fillers. In fact, hydrogels have a unique capability to uptake larger amounts of water to facilitate the immunological activity of the cells’ characteristics, which is crucial in the wound healing progression [3].

In this review, we will discuss the preparation of hydrogels focusing on those with antimicrobial properties. The different methodologies to obtain hydrogels as a function of their role either as a carrier, or based on, or due to their inherent antimicrobial activity will be illustrated with selected examples. Finally, more recent strategies in which the hydrogels exhibit a response to environmental conditions that allow modulating the antimicrobial activity of the hydrogel will be equally discussed throughout this review.

## 2. Strategies to Fabricate Hydrogels and Their Classification

### 2.1. Fabrication via Physical or Chemical Approaches

**Figure 2:**
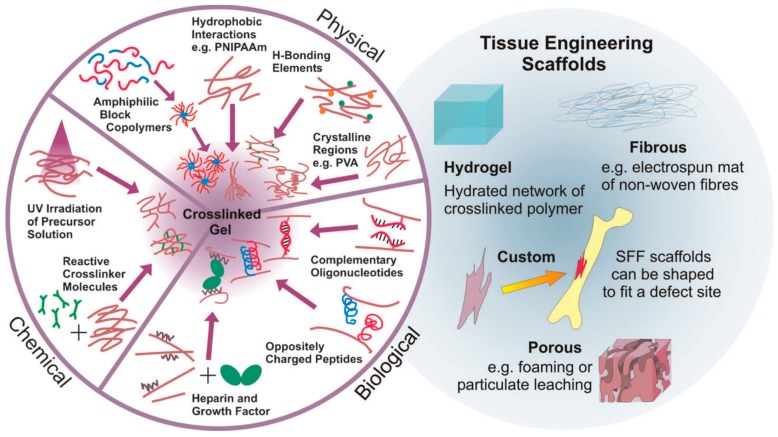
(**Left**): Alternatives to obtain crosslinked hydrogels. Hydrogels can be based on permanent, covalent links between polymer chains or physical (reversible) crosslink bonds based on a variety of non-covalent interactions (from synthetic and biological origin); (**Right**): Examples of TE scaffolds, including hydrogels, fibrous, custom and porous morphologies. Reproduced with permission from [8].

Two main strategies to prepare hydrogels considering the type of bonds formed between the polymer chains have been widely employed. On the one hand, physical hydrogels are formed with non-covalent crosslinks. Thus, the hydrogels can be reversibly formed or disrupted. Non-covalent crosslinks can be, as depicted in Figure 2, of a different nature, including hydrophobic interactions, hydrogen bonds, electrostatic interactions or even crystallized segments. For instance, hydrophobic interactions produced by the self-assembly of amphiphilic block and graft copolymers and further aggregation can finally lead to gelation of the compounds [12]. More importantly, the use of biological molecules for the preparation of physical hydrogels was incorporated later since their particular structures are prone to form larger aggregates by non-covalent interactions. A large variety of biopolymers has been bound to polymer chains enabling the formation of hydrogels by different interactions. As depicted in Figure 2, illustrative biomolecules employed for this purpose include oligonucleotide sequences, peptides with a complementary charge or heparin that binds a multitude of proteins. 

**Figure 3:**
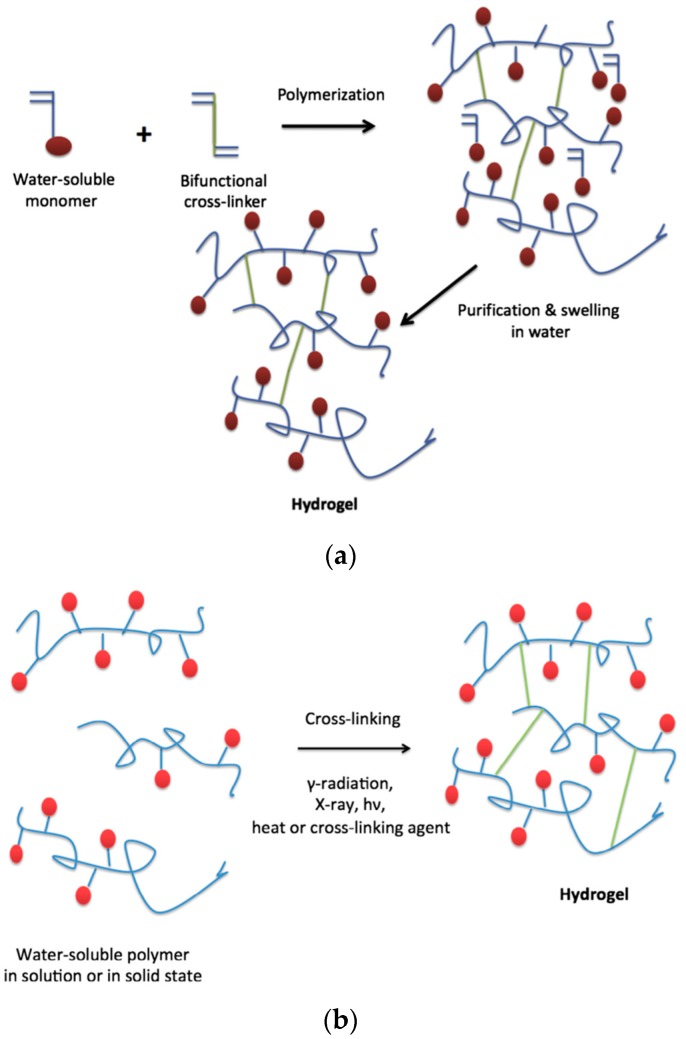
(**a**) Synthesis of hydrogels by polymerization of monomers and crosslinking agent; (**b**) Synthesis of hydrogels by cross-linking of pre-polymerized water-soluble polymers. Reproduced with permission from [16].

On the other hand, chemical hydrogels are formed by covalent crosslinks, in general by the chemical reaction of functionalized polymers or monomers with small molecule/polymer crosslinking agents [13]. Examples of these chemical reactions are shown in Figure 2. These include reactions between thiols and acrylate/vinyl sulfone groups [14] or amines that easily react with aldehydes or activated ester groups [15]. As depicted by Caló and Khutoryanskiy [16], chemical hydrogels are mainly prepared either by a copolymerization of a hydrophilic monomer with the appropriate crosslinking agent that generates strong bonds between polymer chains. As depicted in Figure 3, a water-soluble monomer is polymerized in the presence of a multifunctional crosslinker (typically a difunctional monomer) that, upon a purification step to remove residual unreacted monomer, leads to the final hydrogel. An alternative to this approach is depicted in Figure 3 (below). In this case, instead of using monomers, a preformed water-soluble polymer is crosslinked using, among others, γ-irradiation, X-ray, UV-light or heat. For instance, Khutoryanskiy et al. [17] described a method to fabricate hydrogels from water-soluble polymers (poly(methyl vinyl ether-alt-maleic anhydride) and poly(vinyl alcohol)) in aqueous solutions. To carry out the crosslinking process, they proposed the use of either thermal treatment or microwave irradiation. It is worth mentioning that both radiation and thermal crosslinking methods are inexpensive, safe and permit the elimination of the purification step. 

**Figure 4:**
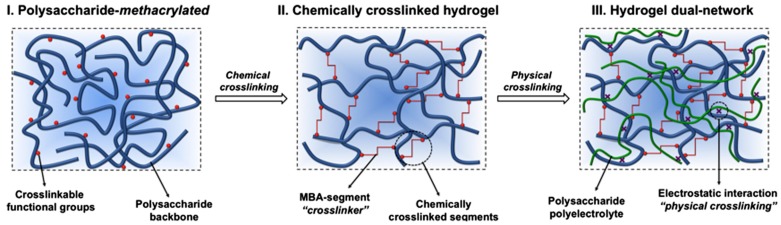
Scheme of dual-network hydrogel formation schematic (Step 1 is the chemical crosslinking; Step 2 is the physical crosslinking). Reproduced with permission from [18].

It is worth mentioning that a third class of hydrogels has been reported combining both types, i.e., physical and chemical crosslinking hydrogels. These are known as dual-network hydrogels. An illustrative example of this type of hydrogel has been reported by Fajardo et al. [18]. As depicted in Figure 4, they described the formation of a novel type of hydrogel that combines chemically- and physically-crosslinked networks in a dual-network approach. For that purpose, chitosan (CHT) and chondroitin sulfate (CS) were chemically modified with glycidyl methacrylate (GMA) and then crosslinked.

### 2.2. Other Alternative Hydrogel Classifications

**Figure 5:**
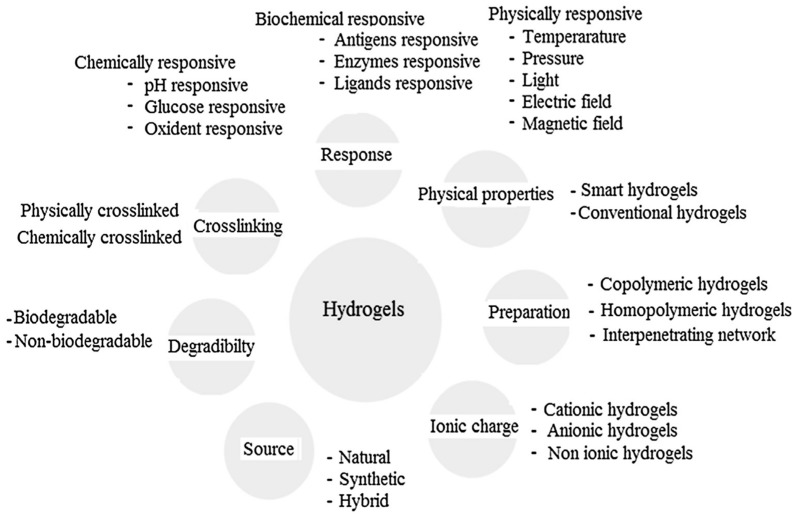
Classification of hydrogels based on the different properties. Reproduced with permission from [13].

Other classifications have been equally proposed for hydrogels attending to their characteristics as the type of crosslinking agent, their eventual response to external stimuli (physically-, chemically- or biochemically-responsive), their net charge (depending on the functional groups incorporated in the hydrogel structure, they can be negative, positive or neutral), their origin (either from natural sources or obtained following a synthetic route) or based on their degradability or the non-degradability of the hydrogel [4,13]. An overview of the different hydrogel classifications depending on their properties is provided in Figure 5.

## 3. Antimicrobial and Antifouling Hydrogels

Bacterial infection remains one of the most serious complications for human health and may possibly lead to death in some severe cases. For this reason, extensive efforts have been conducted toward finding novel antibiotics with low toxicity to the host cell and a broad spectrum against a wide diversity of bacterial pathogens that do not produce bacterial resistance. Antimicrobial agents and peptides alone, however, usually suffer from environmental toxicity, short-term antimicrobial activity and proteolytic instability and degradation. To overcome such disadvantages, antimicrobial agents are often physically incorporated or chemically conjugated with biocompatible polymers, such as hydrogels, to enhance their antimicrobial efficacy and specificity, reduce their cytotoxicity, prolong their biostability and biocompatibility and finally promote other biomimetic physicochemical properties. 

Hydrogels with antimicrobial properties have been obtained using different strategies (described in the following sections) that can be summarized into two main methodologies. First, hydrogels can be employed as supports to load the antimicrobial molecules. As depicted in Table 1, these can be of a different nature, such as nanoparticles (typically gold or silver), antibiotics or antimicrobial agents. Second, the hydrogels can be designed based on materials that present inherent antimicrobial activity [1]. The most relevant examples of inherently antimicrobial materials employed to fabricate hydrogels include antimicrobial peptides, chitosan or synthetic polymers with antimicrobial functional groups (quaternary ammonium, N-halamines, etc.). In addition, in Section 6, the combination of antifouling and antimicrobial polymers to synergistically prevent bacterial contamination is discussed.

## 4. Hydrogels Supporting Antimicrobial Agents

This section aims to provide a thorough overview of the antimicrobial hydrogels prepared by encapsulation or immobilization of antimicrobial compounds incorporating a few selected examples of the different strategies reported. 

### 4.1. Incorporation of Metal Nanoparticles to Hydrogels

Silver and gold nanoparticles (NPs) are the most extensively employed nanoparticles for biorelated applications. This is, at least to some extent, due to the unique antimicrobial properties they exhibit [42,43,44,45,46]. In addition to these, nanoparticles zinc oxide nanoparticles have also evidenced excellent antibacterial properties [43,47]. A comparative study has been reported by Hernández-Sierra et al. [43] in which both the bactericidal and bacteriostatic properties of silver, zinc oxide and gold nanoparticles were investigated over *S. mutans*. In spite of the good properties of the three nanoparticles studied, the authors evidenced a higher antimicrobial effect against *S. mutans* when using silver nanoparticles. Thus, lower concentrations than in the case of gold or zinc nanoparticles were required. This is an important factor that would permit reaching a reduced toxicity.

Both synthetic and natural hydrogels have been employed as supports for the incorporation of nanoparticles [3]. For instance, synthetic hydrogels loaded with metal nanoparticles include poly(*N*-vinyl pyrrolidone) (PVP) [48], poly(vinyl alcohol) (PVA) [19] or poly(acrylamide-co-acrylic acid) [49]. Natural polymers, such as natural gelatin [50] and alginate [20] hydrogels, have been also employed to encapsulate silver nanoparticles. 

Different methodologies have been reported to incorporate nanoparticles with antibacterial properties within a hydrogel being the most important with respect to the following.

#### 4.1.1. Loading Nanoparticles onto a Preformed Hydrogel

**Figure 6:**
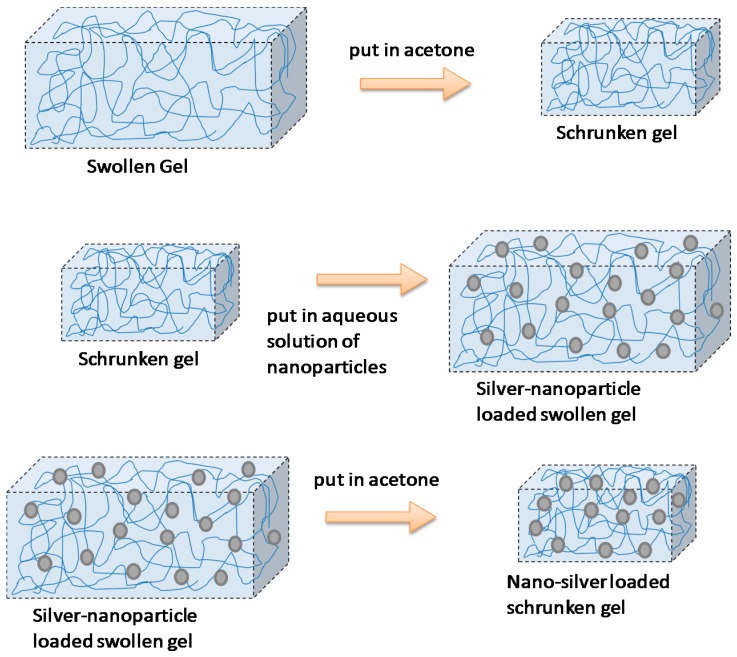
Encapsulation of silver nanoparticles within a hydrogel following the breathing-in/breathing-out (BI-BO) methodology. Reproduced with permission from [51].

This strategy was reported by Thomas et al. [51] to prepare poly(acrylamide-co-*N*-vinyl-2-pyrrolidone) hydrogels loaded with silver NPs (Figure 6). They called this technique the breathing-in/breathing-out (BI-BO) method. The idea behind this BI-BO process relies on alternating changing solutions that can cause the hydrogel to swell and shrink. Therefore, during an initial swelling process, the NPs are diffused into the hydrogel. In a second shrinking process step, the particles are encapsulated into the hydrogel network. The crosslinking degree and the NPs’ content had a great influence on the antibacterial efficacy. Thus, the number of BI-BO cycles is intimately related with the antibacterial activity; in fact, an optimal bactericidal activity is reported after three cycles against *Escherichia coli.*

#### 4.1.2. Formation of the Hydrogel in the Presence of NPs

**Figure 7:**
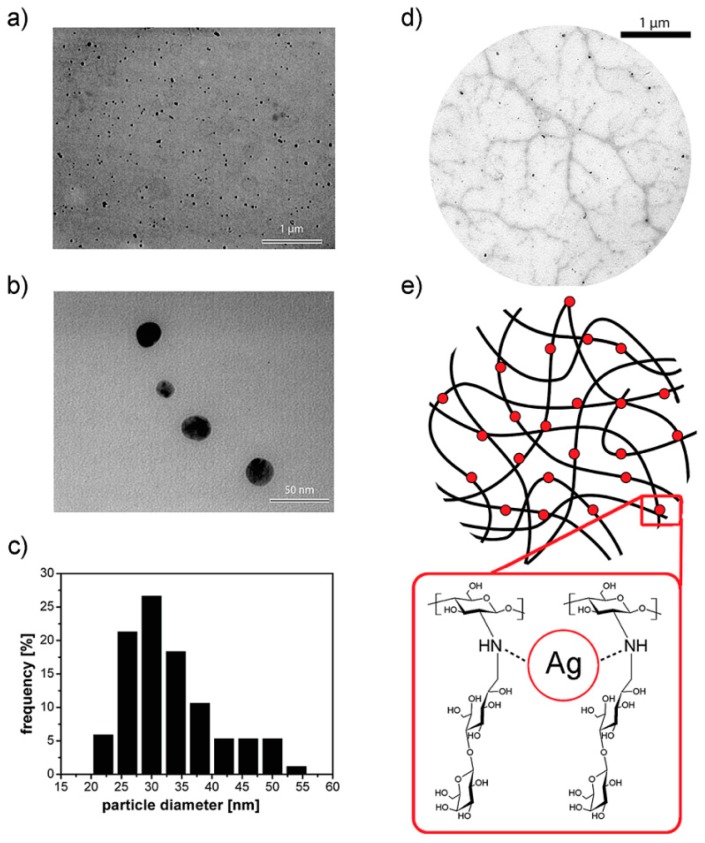
(**a**,**b**) Transmission electron microscopy (TEM) images of silver nanoparticles embedded in a 1-deoxylactit-1-yl chitosan (Chitlac) solution obtained at different magnifications; (**c**) silver nanoparticles size distribution histogram based on the TEM image in (**b**); (**d**) TEM image of silver nanoparticles formed on the polymeric chains of Chitlac; (**e**) schematic representation of the polymeric chains of Chitlac and its interaction with Ag. Reproduced with permission from [52].

This methodology, employed by Yu et al. [48], is based on the incorporation of nanoparticles into hydrogels by using freeze-thaw cycles. They described the preparation of poly(vinyl alcohol)/poly(*N*-vinyl pyrrolidone) (PVA-PVP) hydrogels bearing silver nanoparticles by using this approach. According to the authors’ finding, this methodology permitted the fabrication of hydrogels without significant aggregation of the silver nanoparticles. Furthermore, Travan et al. [52] took advantage of this approach to incorporate silver NPs in the presence of a bioactive chitosan-derived polysaccharide solution. This solution is a lactose-substituted chitosan, 1-deoxylactit-1-yl chitosan, named “Chitlac” (depicted in Figure 7). Once the nanoparticles have been dispersed in the Chitlac solution, the latter was mixed with an alginate solution, thus forming the final hydrogel. The role of the branched polysaccharide Chitlac is crucial in the formation and stabilization of well-dispersed silver NPs.

#### 4.1.3. Fabrication of Hydrogels and Nanoparticles Simultaneously

**Figure 8:**
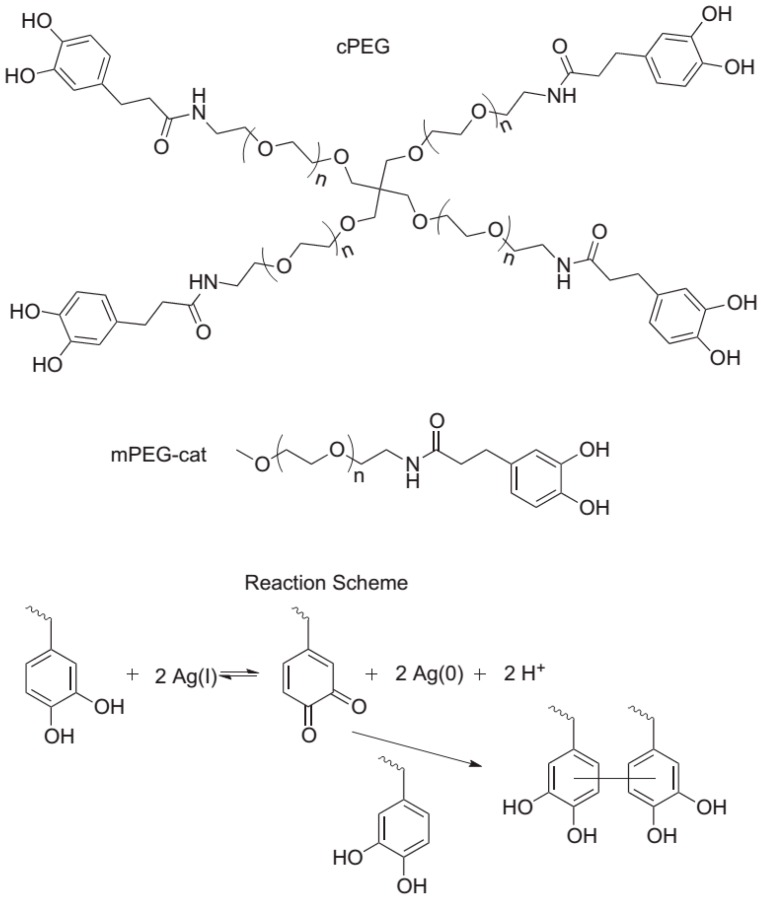
Molecules employed and reaction scheme for the fabrication of composite antibacterial hydrogels in one single step. branched catechol-derivatized poly(ethylene glycol) cPEG was used to form hydrogels and mono catechol-derivatized poly(ethylene glycol) mPEG-Cat was used for model gel permeation chromatography (GPC) studies of the reaction. Catechol reduction of Ag(I) allows for quinone-initiated radical coupling to catechols, as well as simultaneous silver nanoparticle formation. Reproduced with permission from [21].

Fullenkamp et al. [21] employed this strategy for the fabrication of a silver-releasing antibacterial hydrogel. In their strategy depicted in Figure 8, the antibacterial hydrogel simultaneously permits the silver nanoparticle formation and gel curing. For that purpose, they synthesized water-soluble polyethylene glycol (PEG) polymers bearing reactive catechol moieties. Silver nitrate (precursor of the silver nanoparticles) oxidizes the catechol groups leading to covalent cross-linking. Interestingly, this oxidation and, thus, hydrogel formation occur simultaneously with a reduction of Ag(I). The resulting hydrogels were found to inhibit bacteria growth, without significantly affecting the mammalian cell viability.

In addition, Gonzalez-Henriquez et al. [53,54] also generated in situ nanoparticles embedded into a hydrogel matrix acting as a container and stabilizer during the nanoparticle formation. This method was realized using AgNO_3_ as the silver source. Additionally, hydroxyl ethyl methacrylate monomer (HEMA), crosslinking agents diethylene glycol methacrylate (DEGDMA) and polyethylene glycol diacrylate (PEGDA) and a photoinitiator (Irgacure 651 or 2959) were used for the hydrogel synthesis. The reaction mixture was irradiated with a lamp, UV = 365 nm, forming the hydrogel and the Ag^+^ reduction simultaneously. This procedure enhances the stability of the silver NPs and improves the spatial dispersion, as well. Quantitative assays show that certain types of hydrogel present an important biocide property, by reducing 99.9% of bacterium *E. coli*.

Finally, as a summary, it is worth mentioning that, independent of the fabrication approach employed (depicted above), two crucial aspects need to be considered in the preparation of antimicrobial hydrogels using nanoparticles. The first aspect involves the nanoparticle dispersion. The most extended approach to improve the nanoparticle dispersion resorts to the modification of the nanoparticle surface functionality and a high spatial dispersion to avoid the agglomeration of the metallic particles. As a result, the interactions between the hydrogel and the nanoparticles are improved, enhancing the interaction of the material with its surrounding environment, where the bacteria are located [55]. The second important aspect that requires consideration is the cytotoxicity associated with the nanoparticles employed. The different alternatives reported aim to reduce the nanoparticles cytotoxicity to mammalian cells while maintaining antimicrobial activity. Das et al. [56] proposed the use of the counterion of positively-charged hydrophilic hydrogelators. In particular, they evidenced a direct relation between the counterion of a positively-charged amino acid-based hydrogelator (able to change from chloride to a hydrophobic carboxylate) and the minimal inhibitory concentration (MIC) when tested against Gram-positive bacterial and fungal strains. Das et al. found that the MIC for Gram-positive *B. subtilis* decreased from 10.0 down to 2.0 μg/mL when the chloride was exchanged for n-hexanoate. More interestingly, they found that the toxicity towards HepG2 and NIH3T3 mammalian cells also decreased to a large extent.

### 4.2. Hydrogels Loaded with Antibiotics and Antimicrobial Agents

Hydrogels, most of them having a hydrophilic nature, are able to load low molecular weight antibiotics and antimicrobial agents into their network. Some examples of antibiotics that have been incorporated within hydrogels include ciprofloxacin [25,26,57], gentamicin [58], teicoplanin [24] and amoxicillin [59]. Other strategies have been proposed in which the hydrogels were designed to deliver broadly-acting antimicrobial agents that, in contrast to antibiotics, do not develop antimicrobial resistance [27,28,29,60,61,62,63,64].

**Figure 9:**
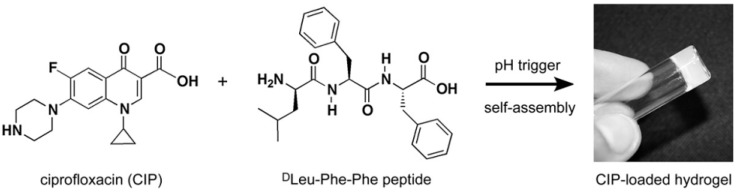
Chemical structures of both ciprofloxacin (CIP) and tripeptide ^D^Leu-Phe-Phe; these structures are able to self-assemble to form a hydrogel following a pH trigger. Reproduced with permission from [25].

Marchesan et al. [25] used ciprofloxacin antibiotics to fabricate antimicrobial hydrogel. These were constructed by self-assembly of ciprofloxacin with the hydrophobic tripeptide (^D^Leu-Phe-Phe). The structures of the hydrogel components are shown in Figure 9. Interestingly, the drug is incorporated within the hydrogel by non-covalent interactions and provides an excellent means to retain the antimicrobial activity of the hydrogel over a prolonged release timescale [25,57].

**Figure 10:**
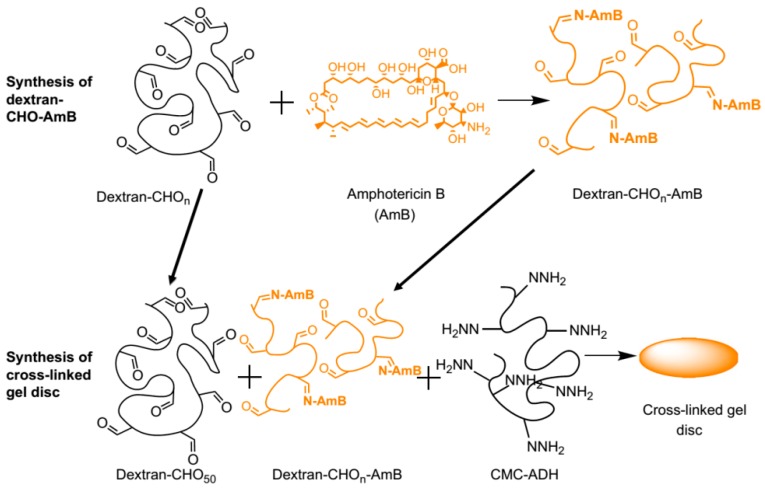
Conjugation of amphotericin B (AmB) to oxidized dextran and the incorporation of the dextran-CHO-AmB into a carboxymethylcellulose (CMC)-dextran gel. Reproduced with permission from ref. [29].

Antimicrobial agents such as amphotericin B (AmB) have been also incorporated into hydrogels. An illustrative example of this strategy was reported by Hudson et al. [29]. As shown in Figure 10, they prepared dextran hydrogels bearing AmB covalently anchored by the reaction between the amine groups present in the AmB and the aldehyde groups provided by the dextran molecule. Injectable systems of this type of compounds could be used as a treatment for local antifungal infections.

## 5. Hydrogels with Inherent Antimicrobial Properties

In addition to the strategies depicted above to fabricate antimicrobial hydrogels by charging the hydrogels with different antimicrobial molecules (drugs, antibiotics, etc.) in a non-covalently manner, polymeric materials with inherent antimicrobial properties have been proposed to overcome some of their limitations. In particular, the effective lifetime of hydrogels loaded with nanoparticles or antimicrobial compounds is rather limited and directly depends on the drug diffusion time. Herein, we will limit our discussion to three of the most extended inherent antimicrobial polymers employed in the elaboration of hydrogels, i.e., synthetic polymers, antimicrobial peptides and natural polymers, focusing on the use of chitosan.

### 5.1. Synthetic Hydrogels with Inherent Antimicrobial Properties

**Figure 11:**
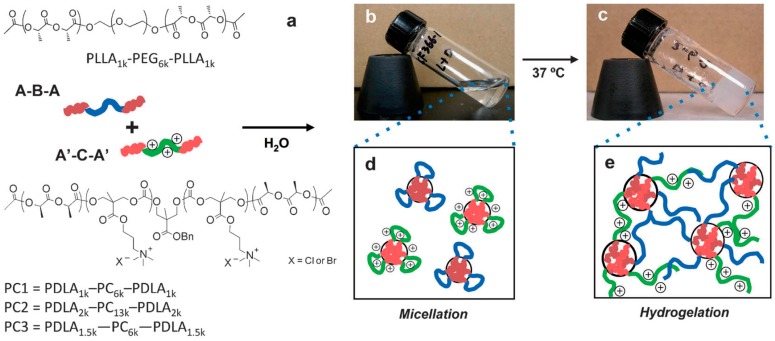
Chemical structures of the mixed micelle solution containing poly(l-lactide)-*b*-poly(ethylene glycol)-*b*-poly(l-lactide) (PLLA-PEG-PLLA) and poly(carbonate)-*b*-poly(d-lactide) (PDLA-CPC-PDLA). Schematic illustration (**a**) and pictures of 10% *w*/*v* solution at 25 °C (**b**) and at 37 °C (**c**). At 25 °C, the solution is a clear fluid, and each polymer forms flower-type micelles in an aqueous environment (**d**). Upon heating at 37 °C for 30 min, the solution turns into an opaque gel based on stereocomplex formation between enantiomeric pure polylactide segments in the micelle cores (**e**). Reproduced with permission from [41].

As reported by Li et al. [41], there is an insistent need to further develop novel antimicrobial hydrogels from synthetic well-defined molecular structures and biodegradable as well as cost-effective materials. Further requirements include the possibility of be moldable/processable, particularly interesting for in situ applications. In order to fulfill these requirements, Li et al. [41] described an effective approach of producing charged hydrogels using noncovalent interactions, thus overcoming many of the issues plaguing existing antimicrobial hydrogels. They fabricated stimulus-responsive antimicrobial gels formed from stereocomplexation of biodegradable poly(l-lactide)-*b*-poly(ethylene glycol)-*b*-poly(l-lactide) (PLLA-PEG-PLLA) and a charged biodegradable polycarbonate triblock polymer (i.e., poly(carbonate)-*b*-poly(d-lactide) (PDLA-CPC-PDLA)) (Figure 11). The stereocomplexes were found to exist as soluble micelles at room temperature in aqueous solution; however, upon heating to physiological temperature (ca. 37 °C), gel-like materials were formed. More interestingly, this drastic change in the material properties is accompanied by a significant increase in antimicrobial activity against Gram-positive/Gram-negative bacteria, fungi and microbial biofilms.

### 5.2. Hydrogels Based on Antimicrobial Peptides 

Synthetic cell-adhesive polypeptide hydrogels with inherent antibacterial activity have been explored to facilitate cell adhesion, proliferation and/or differentiation, in particular for wound healing applications. In general, antimicrobial peptides (AMPs) have a cationic net charge and amphiphilic structure and are, thus, able to establish interactions with the negatively-charged cell membrane [65].

Illustrative examples of employing polypeptides to fabricate antimicrobial hydrogels have been reported by Schneider and coworkers [31,66], Song et al. [33] or Zhou et al. [32]. For instance, in the work of Schneider et al., they developed a family of self-assembling β-hairpin peptides able to form hydrogel networks. The composition of the peptides could be varied from a lysine-rich amphiphilic peptide (i.e., MAX1) [32,33,67] to an arginine-rich polypeptide [68,69,70]. More importantly, these AMPs are very efficient to combat multidrug-resistant bacteria, MRSA [30]. A precise AMP composition (i.e., the peptide containing six arginine residues, PEP6R [31]) was reported to be highly efficient to eradicate bacteria, such as *S. aureus*, *E. coli* and *P. aeruginosa*, while remaining non-cytotoxic towards mammalian cells. In contrast to Schneider, Song et al. [33] fabricated hydrogels using a series of polypeptides poly(lysine-co-alanine) (Lys)_x_(Ala)_y_ crosslinked with six-arm PEG-amide succinimidyl glutarate. This approach equally permitted the variation of the relative amount of alanine and lysine in the compound. As a result, the authors evidenced that poly(Lys)_60_(Ala)_40_ showed selective antibacterial properties, i.e., they are able to kill *E. coli* and *S. aureus* bacteria while exhibiting superior mammalian cell adhesion and cell proliferation activities. Finally, Zhou et al. [32] employed epsilon-poly-l-lysine-graft methacrylamide (EPL-MA) to fabricate antimicrobial hydrogels. The antimicrobial activity of the EPL-MA hydrogels immobilized onto plastic surfaces was evaluated, and these studies established that these hydrogels are broadly active against bacteria and fungi, for example, *C. albicans*, *P. aeruginosa*, *S. aureus*, *E. coli* and *F. solani*.

### 5.3. Natural Polymers with Inherent Antimicrobial Properties

Amongst the different available natural polymers including collagen [61], chitosan is, without any doubt, the most extensively explored polymer for the elaboration of antimicrobial hydrogels. Chitosan is a biocompatible and biodegrade linear polysaccharide able that exhibits low toxicity, with a hydrophilic nature and with low production costs [71,72,73]. Moreover, since chitosan is weakly basic, its amino groups are readily protonated in an acidic medium, enabling the interaction with the bacterial cell membrane, therefore providing exceptional antimicrobial properties [37,74].

Chitosan has been used as the base for the elaboration of multiple antimicrobial hydrogel systems. Selected examples of the strategies explored using chitosan as an antibacterial are depicted below [74]. 

#### 5.3.1. Chitosan Immobilization at the Surface of a Particular Material

For instance, Chen et al. [75] immobilized chitosan onto poly(*N*-isopropylacrylamide) (PNIPAAm) gel/polypropylene (PP) nonwoven composites. They first activated the surface and applied UV light to produce the graft polymerization of NIPAAm gel at the PP surface. In a second step, chitosan was anchored at the surface using the cross-linking agent, glutaraldehyde (GA). These hydrogels were tested against *E. coli* and *S. aureus*, displaying antibacterial activity. 

#### 5.3.2. Chitosan Modification by Using the Amino Side Groups Present along the Main Chain

**Figure 12:**
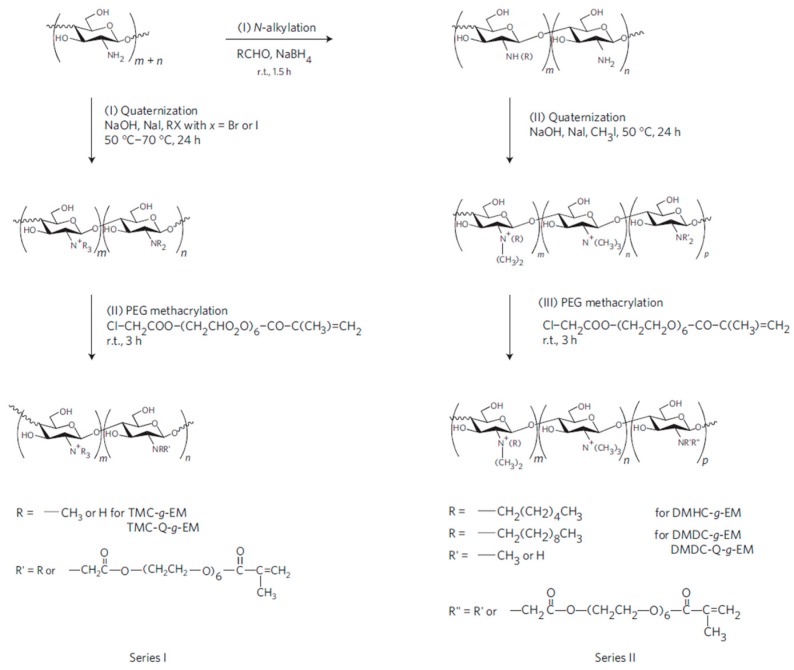
Scheme of the synthesis for the fabrication of modified chitosan antimicrobial hydrogels. Trimethylammonium chitosan-g-poly(ethylene glycol) methacrylate (TMC-g-EM), dimethylhexylammonium chitosan-g- poly(ethylene glycol) methacrylate (DMHC-g-EM) and dimethyldecylammonium chitosan-g- poly(ethylene glycol) methacrylate (DMDC-g-EM) dimethyldecylammonium chitosan (with high quaternization)-graft-poly(ethylene glycol) methacrylate (DMDC-Q-g-EM) Reprinted with permission from [76].

The chemical modification of the amino side groups can be employed to quaternize them, thus improving the antimicrobial properties of chitosan. The approach developed by Li et al. [76] to synthesize antimicrobial hydrogels based on quaternized ammonium chitosan-*graft*-PEG methacrylate (qC-*g*-EM) with variable alkyl chain lengths using two different pathways is depicted in Figure 12. The authors establish that the alkyl chain length of the quaternizing agent from trimethylammonium (TM) to dimethyldecylammonium (DMD) plays a key role in the efficacy against Gram-positive *S. aureus*, but not against Gram-negative bacteria *E. coli* or *P. aeruginosa*. 

#### 5.3.3. Encapsulation and/or Immobilization of Chitosan within the Hydrogel Structure

Another interesting strategy to provide antimicrobial properties to inert hydrogels involves the immobilization of chitosan within a hydrogel. This methodology has been employed by Liu et al. [35], Zhao et al. [77] and Noppakundilograt et al. [78]. For instance, in the work of Liu et al., chitosan was immobilized onto temperature-sensitive poly(*N*-isopropylacrylamide/polyurethane (PNIPAAm/PU) hydrogels. The authors examined the antibacterial behavior of the hydrogels before and after grafting. They concluded that after chitosan modification, the hydrogel grafted nonwoven cellulose fabrics demonstrated an antibacterial activity to *S. aureus* and *E. coli* with an antibacterial efficiency of about 80%.

Finally, other strategies employed chitosan as a component in the formation of polyelectrolyte complex hydrogels [79] or to form hydrogels by crosslinking reactions [80,81,82]. 

## 6. Hydrogels with Both Antifouling and Antimicrobial Properties

Another interesting characteristic, besides antimicrobial activity, that would synergistically prevent bacterial contamination is the antifouling property. Several biomedical applications need materials that possess low fouling capacity. In general, polymeric materials are prone to protein adsorption due to their hydrophobic nature. Protein fouling can occur in few seconds after implantation and exposure to body fluids, resulting in blood clots and subsequent thrombosis. Once the proteins form the topmost layer on the surface, microbes, such as bacteria and fungi, can easily anchor onto the surface, generating a biofilm, finally leading to severe infections.

**Figure 13:**
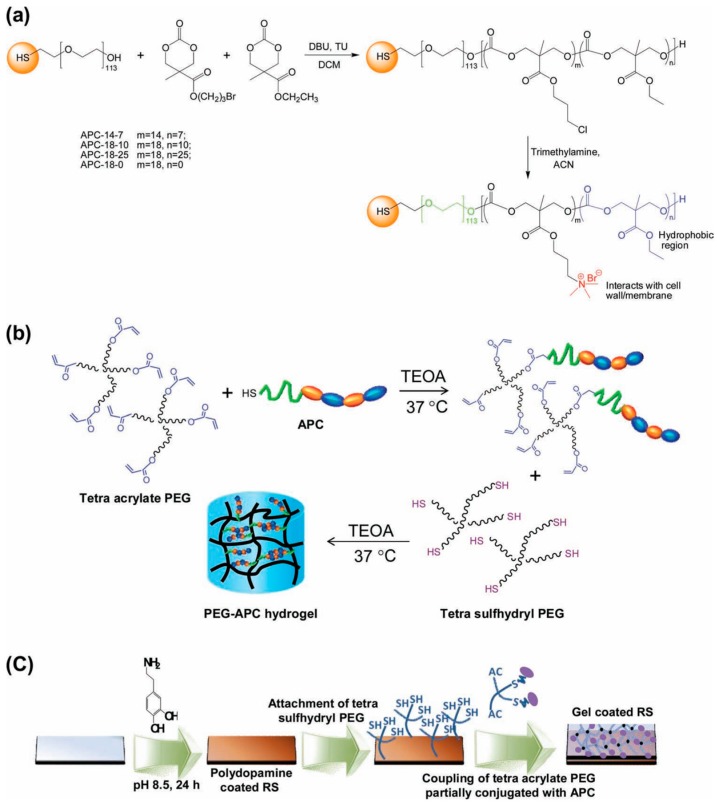
Synthetic scheme for the fabrication of (**a**) aminated polycarbonates (APCs); (**b**) PEG-APC hydrogel; and (**c**) hydrogel coating onto silicone rubber surface. Reproduced with permission from [39]. TEOA: triethanolamine. RS: Silicon rubber.

Several groups reported studies attempting to combine antimicrobial properties with antifouling behavior in order to additionally reduce the initial bacterial attachment to the scaffold [39,40]. An illustrative example of hydrogels combining both properties was reported by Liu et al. [39] who described the fabrication of hydrogels bearing intrinsically antifouling polyethylene glycol (PEG)-based hydrogels combined with polycarbonate groups (polycarbonate containing quaternary ammonium groups, aminated polycarbonate (APC)). The synthesis of these hydrogels is depicted in Figure 13. Briefly, they prepared thiol-terminated block copolymers composed by PEG and cationic polycarbonate segments fabricated via an organocatalytic ring opening polymerization (ROP) at room temperature. The amount of randomly-distributed hydrophobic ethyl groups and quaternary ammonium groups could be thus easily varied (Figure13a). The cationic PEG-APC linear polymers were then turned into hydrogels by using a tetra-acrylate functional PEG that reacted with the thiol-containing APC. As a result, a solution of star-shaped PEG, which was partially conjugated with APC, was obtained, then the addition of a stoichiometric amount of tetra-sulfhydryl PEG results in gelation within a few minutes (Figure 13b). Finally, in order to mimic a catheter surface, the gel was supported on silicone rubber. It is worth mentioning that these hydrogels evidenced an effective synergistic antifouling and antimicrobial activity both against *S. aureus* and *E. coli*.

Another example of a simultaneously antifouling and antimicrobial hydrogel was reported by Zhao et al. [83]. They fabricated hybrid poly(*N*-hydroxyethyl acrylamide) (polyHEAA)/salicylate (SA) hydrogels and tested them, attempting to identify their antifouling and antimicrobial properties separately. On the one hand, the antifouling efficacy of polyHEAA hydrogels was evaluated exploring the initial adhesion of proteins, cells and bacteria. On the other hand, the authors evaluated the antimicrobial activity of polyHEAA/SA hydrogels against both Gram-negative (*E. coli*) and Gram-positive (*S. epidermidis*) bacteria. They concluded that the polyHEAA/SA hydrogels displayed high surface resistance to protein adsorption, cell adhesion and bacteria attachment and also presented excellent antibacterial properties against both Gram-negative and Gram-positive bacteria. 

Other recent works reported by Liu et al. [39] and Cao et al. [40] reported the use of synthetic polymers to produce simultaneously antifouling and antimicrobial hydrogels. Liu et al. employed polycarbonate and poly(ethylene glycol) as hydrogel components. Cao et al. developed zwitterionic materials, poly(2-((2-hydroxyethyl)(2-methacryloyloxy)ethyl)(methyl) ammonio)acetate (pCBOH1) and poly(2-(bis(2-hydroxyethyl)(2-(methacryloyloxy)ethyl ammonio)acetate) (pDBO112), to form the hydrogels. These materials are, under neutral or in basic conditions, in their zwitterionic form, exhibiting an ultra-fouling behavior. On the contrary, in acidic conditions, they are positively charged and exhibit antimicrobial properties. 

## 7. Design of Antimicrobial Hydrogels with Stimuli-Responsive Polymers 

The concept of stimuli-responsive hydrogels refers to water-swollen, chemically- or physically-crosslinked compounds that can undertake large changes in volume or shape as a result of a slight variation of the environmental conditions [84,85,86,87]. These hydrogels have been employed for different purposes, including actuators [88,89,90], as sensors [91], or in biomedical applications, such as tissue engineering applications [92], or as scaffolds for cell culture [5].

The use of stimuli-responsive polymers has been also extended to the fabrication of antimicrobial hydrogels. Two main advantages have been reported in the use of stimuli-responsive macromolecules in the preparation of antimicrobial hydrogels. On the one hand, under certain conditions, the antimicrobial agent can be protected from the environment [59]. On the other hand, it is possible to take advantage of changes in the environment to deliver a particular antimicrobial agent [93].

Different hydrogels that respond to pH, temperature, electric fields or even combining responses to different stimulus have been described in the recent literature (Table 2). Hydrogels that respond to changes in the environmental pH have been, for example, reported by Chang et al. [59]. They described the fabrication of chitosan/poly-γ-glutamic acid nanoparticles incorporating amoxicillin to provide pH-sensitive hydrogels as efficient carriers for amoxicillin delivery. The prepared hydrogels allow for drug protection during transport, which is still a crucial issue when using antibiotics. The pH-sensitive hydrogels protect the nanoparticles from being destroyed by gastric acid. As a result, amoxicillin-loaded nanoparticles embedded within the hydrogel are protected and permitted amoxicillin interaction precisely with intercellular spaces, the site of *H. pylori* infection. 

**Figure 14:**
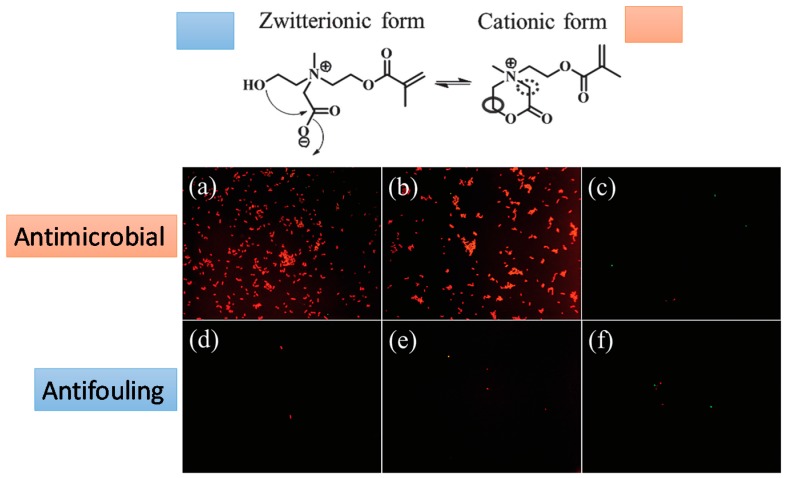
Fluorescence microscopy images of bacterial attachment on pCBOH1 in cationic form (**a**); pCBOH2 in cationic form (**b**); and poly(carboxybetaine methacrylate) pCBMA (**c**) hydrogels before hydrolysis and on pCBOH1 in zwitterionic form (**d**); pCBOH2 in zwitterionic form (**e**); and pCBMA (**f**) hydrogels after 16 h of hydrolysis in PBS. Cells with damaged cytoplasmic membrane are in red, and cells with intact cytoplasm membrane are in green. Reproduced with permission from [40].

Changes in the environmental pH can induce in particular hydrogels changes in their behavior between antifouling and antimicrobial. A smart system with these properties was elaborated by Cao et al. [40]. They fabricated hydrogels based on poly(2-(bis(2-hydroxyethyl) (2-(methacryloyloxy) ethyl) ammonio) acetate) (pCBOH2) and poly(2-((2-hydroxyethyl) (2-(methacryloyloxy)ethyl) (methyl) ammonio) acetate) (pCBOH1). As depicted in Figure 14, in acidic media, these hydrogels become cationic and are able to bind to and kill bacteria. Interestingly, once the material has killed the bacteria, a change in environmental pH back to neutral or basic conditions converts the material to its zwitterionic form, and the dead bacteria can be now released from the hydrogel. In summary, these antimicrobial hydrogels are able both to kill bacteria and prevent the accumulation of dead bacteria at the hydrogel surface.

Thermosensitive polymers, mainly polymers or copolymers of poly(*N*-isopropylacrylamide) (PNIPAm), have been widely used in the fabrication of thermoresponsive coatings [99,100,101,102]. In order to provide antibacterial properties to the thermoresponsive hydrogels, these are generally combined with antibacterial monomers. For instance, Chen et al. [96] and Dizman et al. [97] reported the fabrication of quaternized methacrylamide (MA) combined with NIPAm. At low temperature, these polymers were soluble, and high levels of antibacterial activity against both *S. Aureus* and *E. coli* were observed. This antibacterial activity is caused by the interaction of the polymer quaternary groups and the bacteria [96,97]. By increasing the temperature above the lower critical solution temperature (LCST), the polymers become insoluble, and the antimicrobial activity disappeared [97].

Finally, also electrically-responsive anti-adherent hydrogels, as well as hydrogels that respond to more than one stimulus have been fabricated. On the one hand, the presence of ions diffusing in the hydrogels (known as “iontophoresis”) applying an electric field was employed by Fallows et al. [63] to finely tune the delivery and therefore the antimicrobial activity of a particular hydrogel. Finally, incorporating different sensitive polymers to more than one stimulus can synergistically improve the antibacterial properties of the hydrogel. An illustrative example of this approach was reported by Sui et al. [98] that combined in the same hydrogel monomers that respond to temperature, PNIPAm with redox-responsive poly(ferrocenylsilane) (PFS) macromolecules. Interestingly, the redox activity of the PFS chains in the hybrid hydrogels could be used to prepare PFS-PNIPAM-Ag composites in a facile in situ process. These composites presented strong antimicrobial efficiency against *E. coli* while maintaining a high biocompatibility with cells. 

## 8. Hydrogel Toxicity

According to the fact that several biomedical applications are described in this review, hydrogel toxicity must be included. This section describes important aspects that must be considered before choosing a particular hydrogel for a specific biomedical purpose. As a first example, studies of in vitro cytotoxicity of hydrogel-carbon nanotubes-chitosan (hydrogel-CNT-CH) composites on intestinal cells were presented by Bellingeri et al. [103]. These biomaterials are a promising system for drug delivery applications because this composite does not generate alterations in cellular redox equilibrium, toxicity or apoptosis; important characteristics to consider at the moment of designing a biomedical device. 

Another example are injectable thermosensitive hydrogels that provide local non-invasive platforms for sustained drug release, tissue engineering and cellular immunity. Dong et al. developed a novel self-supported thermosensitive hydrogel based on poly(ε-caprolactone-co-1,4,8-trioxa[4.6]spiro-9-undecanone)-poly(ethylene glycol)-poly(ε-caprolactone-co-1,4,8-trioxa[4.6]spiro-9-undecanone) (PECT) copolymer nanoparticles for locoregional paclitaxel delivery [104,105]. These studies demonstrated that the subcutaneously injected PECT hydrogel only provoked a mild inflammation response. Thus, when PECT was completely absorbed, the subcutaneous tissue would recover itself, and mature dermal fibrous tissue is formed. Additionally, the studies of the toxicity and in vivo biological effect assessments of an amphiphilic copolymer PECT nanoparticles and its self-supported hydrogel were evaluated by the same research group. In vitro cytotoxicity indicated that no cell cytotoxicity was observed when the concentration of PECT nanoparticles was up to 500 µg/mL, and also, no mutagenic effect and no genotoxicity were observed. In vivo intravenous injection of PECT nanoparticles demonstrated that the LD_50_ was approximately high at 2.564 g/kg compared with the control mice. The mice treated with daily the administration of PECT nanoparticles showed no differences in physical or behavioral alterations, body weight changes, biochemical and hematological parameters, nor organ coefficients. The in vivo chronic effect of PECT NP gel confirmed no toxic lesions to animals in a whole period of three months even when the dosage was high at 20 g/kg. These findings indicated that PECT nanoparticles and PECT NP gel were highly biocompatibility and did not provoke any side effects on body, which represented a new class of injectable and non-invasive systemic or site-specific delivery carriers [106].

Another interesting example to consider about toxicity is that explained by Bartholomay et al. [5] about mosquito-borne disease. This disease continues to remain a major threat to human and animal health and also arises as an impediment for socioeconomic development. Thus, the increasing mosquito resistance to chemical insecticides is a great public health concern. According to this problem, the use of nanoparticles (NPs) as a platform to deliver nucleic acid-based mosquitocidal molecules was developed. This research group proposed a method based on particle replication in non-wetting templates (PRINT) that was used as the master template fluorescently labeled with polyethylene glycol nanoparticles of specific sizes and charges (negative or positive). The biodistribution, persistence and toxicity of PRINT NPs were evaluated in vitro in mosquito cell culture and in vivo in *Anopheles gambiae* larvae. The excellent low cell and larval toxicity profiles, efficient internalization and widespread biodistribution make these NPs attractive candidates for dsRNA delivery in mosquitoes. The presence of NPs in head and ovaries may be indicators of contact uptake and vertical transmission capabilities, respectively. These attributes could be exploited to control adult, as well as larval mosquitoes [107]. 

## 9. Conclusions

In this manuscript, recent alternative methodologies to fabricate antimicrobial hydrogels have been discussed. In general, the methodologies reported up to date can be grouped into two main strategies. The first strategy is based on the incorporation of the antimicrobial agents (physically or covalently bonded) within the hydrogel. In the case of physically-bonded antimicrobial, the lifetime of the antimicrobial activity will be directly related to the releasing process. The second strategy takes advantage of the use of polymers with intrinsic antimicrobial properties to fabricate the hydrogels. Polymers with inherent antimicrobial properties include, polymeric hydrogels constructed with cationic monomers, antimicrobial peptides or even naturally-occurring chitosan. The opposite charge of the cationic hydrogels related to the bacterial membrane enables the interaction through non-stereospecific mechanisms that finally lead to membrane disruption. Membrane disruption is another interaction mechanism different from what typically antibiotics do and prevents bacteria from gaining resistance while being active against current strains of multidrug-resistant bacteria. 

Another interesting aspect pointed out in this review is the possibility to precisely switch the antimicrobial activity depending on the environmental conditions. We have described how combining inherent antibacterial hydrogels with stimuli-responsive polymeric systems can precisely modulate the conditions in which one antimicrobial polymeric system has to be turned or remain otherwise inactive. 

## Figures and Tables

**Table 1:** Summary of the strategies reported to fabricate antimicrobial hydrogels. Reproduced with permission from [13].

Type of Antimicrobial Gels		Applications	References
**Loaded hydrogels antimicrobials**	Silver NPs	Wound dressings and surface coatings	[19,20,21]
	Gold NPs	Wound dressings	[22,23]
	Antibiotics	Wound dressings and implant coatings	[24,25,26]
	Antimicrobial agents	Wound dressings and surface coatings	[27,28,29]
**Inherently active hydrogels based on**	Peptides	Wound dressings and surface coatings	[30,31,32,33]
	Chitosan	Wound dressings, surgical use and surface coatings	[34,35,36,37,38]
	Synthetic polymers	Surface coatings	[39,40,41]

**Table 2:** Summary of the most relevant stimuli-responsive antimicrobial hydrogels.

Stimulus	Polymers Employed	References
pH	Chitosan/poly(γ-glutamic acid)	[59]
	Poly(acrylic acid) and poly(vinylpyrrolidone)	[94,95]
	Poly(2-(bis(2-hydroxyethyl) (2-(methacryloyloxy) ethyl) ammonio) acetate) (pCBOH2) and poly(2-((2-hydroxyethyl) 2-(methacryloyloxy)ethyl) (methyl) ammonio) acetate) (pCBOH1)	[40]
Temperature	Poly(*N*-isopropylacrylamide) (PNIPAm)/quaternized methacrylamide (MA)	[96,97]
	Quaternized chitosan and alpha, beta-glycerophosphate (alpha,beta-GP)	[93]
	Poly(l-lactide)-bPEG)-*b*-poly((l-lactide) (PLLA-PEG-PLLA), poly(d-lactide)-bPEG)-*b*-poly((d-lactide) (PDLA-PEG-PDLA), and the cationic triblock polymer poly(d-lactide)-b-cationic poly(carbonate)-*b*-poly(d-lactide) (PDLA-CPC-PDLA)	[41]
Electric Field	Polyelectrolyte poly(methyl vinyl ether-*co*-maleic acid) (PMVE/MA) crosslinked with polyethylene glycol (PEG)	[63]
Multiresponsive	Hydrogel with monomers that respond to temperature PNIPA with redox-responsive poly(ferrocenylsilane) (PFS) macromolecules	[98]
